# Correlation Between Anteroposterior/Transverse Ratio of Inferior Vena Cava Diameter on Computed Tomography and Prognosis in Patients With Sepsis: A Single-Center Retrospective Cohort Study

**DOI:** 10.7759/cureus.95880

**Published:** 2025-11-01

**Authors:** Shintaro Shimozawa, Daisuke Usuda, Yuta Hotchi, Ippei Osugi, Kentaro Mishima, Keiko Mizuno, Hiroki Takami, Takayuki Komatsu, Tomohisa Nomura, Manabu Sugita

**Affiliations:** 1 Department of Emergency and Critical Care Medicine, Juntendo University Nerima Hospital, Tokyo, JPN; 2 Department of Sports Medicine, Juntendo University, Tokyo, JPN

**Keywords:** ap/t ratio, computed tomography, inferior vena cava, prognosis, sepsis

## Abstract

Background

Sepsis is a life-threatening condition with high mortality, and accurate early prognostication remains difficult. The anteroposterior-to-transverse (AP/T) diameter ratio of the inferior vena cava (IVC), measured on computed tomography (CT), offers a simple, objective indicator of intravascular volume status and may correlate with outcomes.

Objectives

To assess whether a lower CT-derived IVC AP/T diameter ratio - used as a surrogate for intravascular volume status - is associated with increased 30-day all-cause mortality in adult sepsis patients and to evaluate whether stratification using an optimal cutoff can differentiate mortality risk.

Methods

In this single-center retrospective cohort study, we included adult patients diagnosed with sepsis from April 1, 2020 to April 1, 2023. The IVC AP/T ratio was measured on CT scans just below the renal veins. The primary outcome was 30-day all-cause mortality. A receiver operating characteristic (ROC) analysis identified the optimal cutoff, and patients were stratified accordingly to compare mortality.

Results

Of 435 patients, 396 had evaluable CT data. The overall 30-day mortality rate was 20.6%. Non-survivors had a significantly lower median IVC AP/T ratio than survivors (0.401 vs. 0.525, p<0.001). In multivariable logistic regression incorporating age, serum lactate, components of the Sequential Organ Failure Assessment (SOFA) score, and the IVC AP/T ratio, the AP/T ratio was not identified as an independent predictor of mortality. The receiving operator characteristic (ROC) analysis yielded an area under the curve (AUC) of 0.64, identifying 0.44 as the optimal cutoff. At this threshold, the accuracy was 63.5%, sensitivity 61.7%, and specificity 68.6%. Patients with an AP/T ratio ≤0.44 were found to have significantly higher mortality than those with a ratio >0.44 (33.6% vs. 12.6%, p<0.001).

Conclusions

The IVC AP/T ratio assessed via CT was significantly associated with 30-day mortality in univariate analysis and may help stratify sepsis patients into different risk categories. However, it was not identified as an independent predictor in multivariable analysis, and its discriminatory ability was modest (AUC 0.64). These findings suggest that while the metric is not sufficient as a standalone predictor, it may serve as a supplementary risk indicator. Further prospective, multicenter studies are warranted to validate its clinical utility and integration into existing prognostic models.

## Introduction

Sepsis is defined as a life-threatening organ dysfunction resulting from a dysregulated host response to infection [1]. Both sepsis and septic shock are recognized as highly prevalent and critical conditions associated with substantial mortality rates and significant economic burdens on healthcare systems worldwide [2-4]. The in-hospital mortality rate for sepsis has been reported at 17%, increasing to 26% in severe cases [5]. Extrapolations based on data from high-income countries estimate approximately 31.5 million annual cases of sepsis and 19.4 million cases of severe sepsis, resulting in up to 5.3 million deaths globally each year [5]. The current diagnostic standard is defined by the Sepsis-3 criteria, as outlined in the Surviving Sepsis Campaign: International Guidelines for Management of Sepsis and Septic Shock 2021 (SSCG 2021) [6]. The diagnostic process for sepsis incorporates various screening strategies designed to identify patients at increased risk of mortality [7-10]. Sepsis-3 recommends the Sequential Organ Failure Assessment (SOFA) score as a tool to predict mortality based on organ dysfunction, emphasizing the importance of rapid and accurate initial evaluation for optimal outcomes [6]. Early recognition and correction of intravascular volume depletion are central to sepsis management, with appropriate fluid resuscitation constituting a cornerstone of treatment [6].

Sepsis is frequently encountered in general clinical practice and is often managed by non-specialists [11]. One established method for evaluating fluid responsiveness involves assessing the diameter and respiratory variability of the inferior vena cava (IVC), which reflects intravascular volume status [12-15]. Although IVC evaluation is typically performed using ultrasonography, its accuracy may vary depending on operator expertise and patient-specific anatomical factors [16]. Additionally, it may not be feasible to obtain absolute measurements at the time of admission, necessitating serial evaluations [17].

Computed tomography (CT)-based assessment of the anteroposterior-to-transverse (AP/T) diameter ratio of the IVC enables objective and reproducible measurements, even in non-specialist settings. Compared to absolute IVC diameters, which are highly dependent on patient body size, the AP/T ratio reflects the relative shape deformation of the vessel and may more directly represent collapsibility and volume status. Moreover, this approach allows for a simultaneous identification of the infection source using routine diagnostic imaging [18]. If the IVC AP/T ratio measured by CT correlates with mortality, it may serve as a valuable tool for early risk stratification and fluid status assessment in sepsis, particularly in general clinical settings [18]. Thus, CT-derived IVC indices may offer a practical and scalable method for enhancing sepsis diagnostics and evaluating preload and fluid responsiveness. Previous studies have reported associations between IVC diameter measured by CT and patient prognosis in trauma populations [19-21]. However, to date, no studies have investigated the prognostic value of CT-based IVC assessment specifically in patients with sepsis. Unlike prior studies that focused solely on septic shock patients [18], our study included a broader population of adult patients diagnosed with sepsis according to Sepsis-3 criteria, regardless of shock status.

## Materials and methods

Study design and setting

We conducted a retrospective observational study at the Department of Emergency and Intensive Care Medicine, Juntendo University Nerima Hospital, a 490-bed community hospital in Tokyo, Japan. Adult patients (aged ≥18 years) who were diagnosed with sepsis and admitted between April 1, 2020, and April 1, 2023, were eligible for inclusion. Only patients with complete hospital records were included. The electronic medical records were retrospectively accessed for research purposes after the observation period, on April 1, 2023.

Data collection

Data were extracted from electronic medical records and included demographic, clinical, laboratory, and outcome information. At admission, patients underwent standardized assessment including medical history, physical examination, blood tests (including arterial blood gases), electrocardiography, pulse oximetry, and CT. SOFA scores and relevant prognostic markers were recorded [18]. Patients were followed until discharge. Inclusion criteria were patients aged 18 years or older who were diagnosed with sepsis according to the Sepsis-3 criteria and had available CT imaging that permitted measurement of the IVC. Exclusion criteria comprised patients younger than 18 years, those without CT imaging, or those in whom the IVC could not be adequately identified or measured.

CT acquisition protocol

All CT scans were performed using a multidetector scanner (Aquilion Prime TSX-303B; Canon Medical Systems Co., Tochigi, Japan). Scan parameters included a tube voltage of 120 kVp and automatic tube current modulation. Images were reconstructed using a deep learning-based algorithm (AiCE) with a soft-tissue kernel (BODY_SHARP) and standard intensity (STD). The matrix size was 512×512 pixels, and the typical field of view (FOV) was 320 mm, adjusted up to 500 mm for larger patients. Axial images were obtained with a 5 mm slice thickness and 5 mm reconstruction interval using soft-tissue window settings (window level 50, window width 270). For contrast-enhanced CT, a weight-based iodinated contrast medium (iohexol 300 mgI/mL) was administered at 500 mgI/kg. For example, a 60 kg patient received 100 mL over 70 seconds (1.4 mL/second), with image acquisition at 115 seconds post-injection (equilibrium phase). Both contrast and non-contrast scans were included depending on clinical indication, as the minimal contrast volume and stable hemodynamics were considered to have negligible impact on IVC diameter measurements.

Measurement of IVC

**Figure 1:**
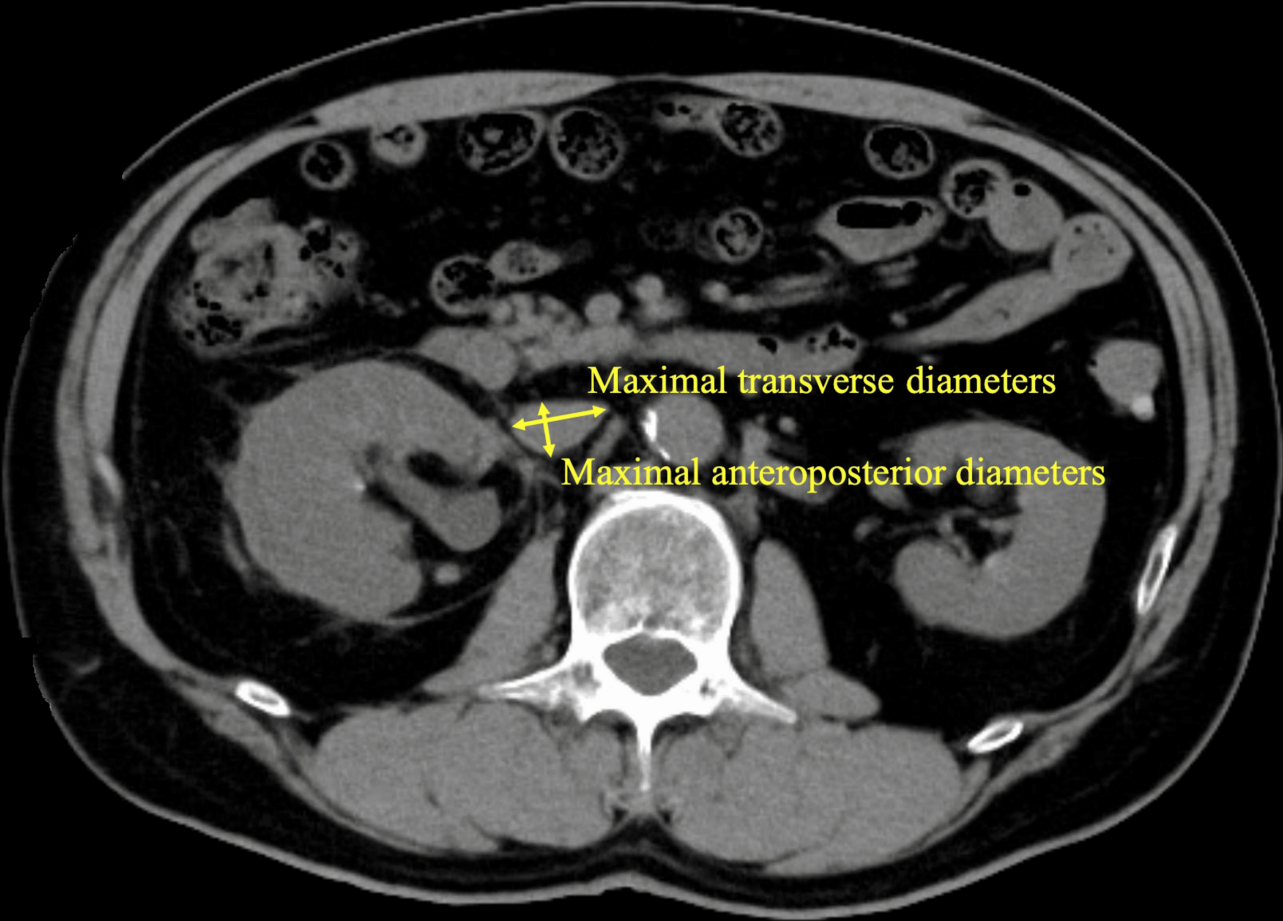
How to measure inferior vena cava (IVC) diameter in CT Measured on axial slice just inferior to the renal veins. AP/T=AP diameter/transverse diameter.

CT images were analyzed on axial views at the level just inferior to the renal veins (Figure 1), chosen for its anatomical consistency and relevance in previous sepsis studies [18,22]. Two senior emergency medicine residents independently and retrospectively measured the IVC diameters using manual calipers on single axial slices. The anteroposterior (AP) and transverse (T) diameters were recorded, and the AP/T ratio was calculated. If the discrepancy between measurements exceeded 5%, consensus was reached through joint review. Inter-rater reliability was assessed using a one-way random effects model, yielding an intraclass correlation coefficient (ICC) of 0.91 (95% CI, 0.85-0.97), indicating excellent agreement. The IVC cross-sectional area was also calculated by manual tracing of the vessel boundary. Image readers were blinded to all clinical data including patient outcomes and timing of CT acquisition relative to fluid resuscitation. IVC diameters were measured manually using the electronic caliper tool within the institution’s Picture Archiving and Communication System (HOPE LifeMark - PACS, version 1.28.00 (64-bit); Fujitsu Ltd., Tokyo, Japan).

Definitions

Sepsis was defined according to the Sepsis‑3 criteria, as a suspected or documented infection accompanied by an acute increase in the SOFA score of ≥2 points from baseline [6]. Infection was determined through comprehensive clinical assessment. All clinical care was provided in accordance with the Surviving Sepsis Campaign: International Guidelines for Management of Sepsis and Septic Shock 2021.

The primary outcome was to evaluate whether stratification of patients based on the CT-derived IVC AP/T ratio cutoff value was associated with differences in 30-day all-cause mortality across groups. The cutoff was determined using the Youden index from the area under the receiver operating characteristic curve (AUROC) analysis to optimize discrimination between survivors and non-survivors.

Statistical analysis

The required sample size (n=352) was estimated a priori based on a previously reported effect size (Cohen's d): 0.30 [18], with α=0.05 and β=0.20. In their cohort, to justify our sample size, we estimated an effect size of Cohen’s d=0.30, based on previously published data reporting IVC AP/T ratios of 0.71 (interquartile range (IQR) 0.54-0.84) in survivors (n=351) and 0.64 (IQR 0.40-0.76) in non-survivors (n=72). Standard deviations were approximated using SD≈IQR/1.35, yielding SDs of 0.222 and 0.267, respectively. The pooled SD was calculated to be approximately 0.230. Assuming the median as a representative value for each group, the effect size was computed. This effect size corresponds to a small-to-moderate difference, which may still have clinical implications for mortality risk stratification in sepsis. Our final cohort (n=396) exceeded this threshold. Continuous variables were summarized as mean±standard deviation or median with interquartile range (IQR), as appropriate, and categorical variables as frequencies and percentages.

Group comparisons were performed using the Student’s t-test or Mann-Whitney U test for continuous variables and the chi-square test for categorical variables, according to data distribution. For multivariable logistic regression, eight covariates were selected a priori based on clinical relevance and incorporation in sepsis severity scores: age, serum lactate, five components of the SOFA score, and the IVC AP/T ratio. This selection was intended to provide adequate adjustment while minimizing overfitting, considering the number of outcome events.

Diagnostic performance was evaluated by calculating the AUROC, accuracy, sensitivity, specificity, positive predictive value (PPV), and negative predictive value (NPV) at the selected threshold. The Youden index was used to determine the optimal cutoff because it maximizes the combined sensitivity and specificity, providing a balanced threshold for distinguishing survivors from non-survivors. A post hoc power analysis was conducted solely for descriptive purposes, confirming that the observed difference in 30-day mortality (33.6% vs. 12.6%) was detectable with >90% power given the available sample size. Missing data were assumed to be at random (MAR) and addressed using complete-case analysis; multiple imputation was also performed as a sensitivity analysis using chained equations. A two-sided p-value <0.05 was considered statistically significant. All analyses were performed using STATA® software (version 10; StataCorp LP, College Station, TX, USA).

Patient and public involvement

Patients and the public were not involved in the design, conduct, or reporting of this retrospective study. Given its nature and use of routinely collected data, direct patient involvement was not feasible. Study information was disclosed on the hospital website, and patients were allowed to opt out, as approved by the ethics committee.

Ethical approval and informed consent

This study was approved by the Institutional Review Board of Juntendo University Nerima Hospital (E23-0109-N01). The IRB approved the opt-out procedure and waived the requirement for written or oral informed consent, as all data were anonymized by hospital data custodians prior to research access. No investigators had access to identifiable patient information at any stage. No minors were included in this study.

## Results

Participant characteristics

**Figure 2:**
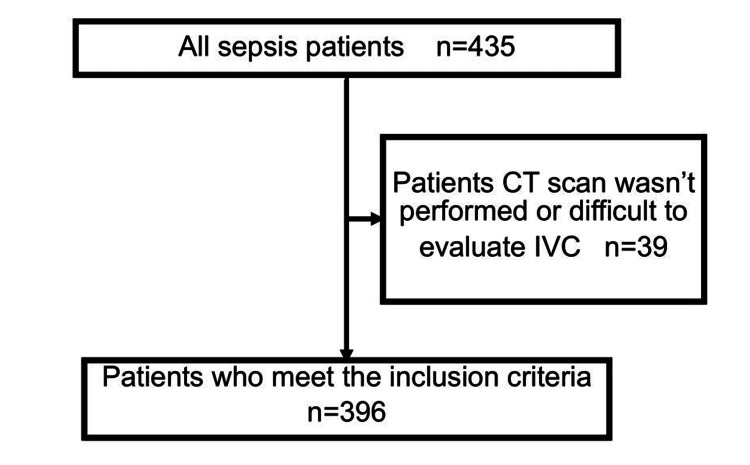
Flow diagram of the study population During the study period, 435 adult sepsis patients were screened via chart review. A total of 39 patients did not undergo CT scans, or it was infeasible to measure the patients’ IVC diameter using CT scans. Ultimately, 396 sepsis patients were included in the analysis.

As shown in Figure 2, a total of 435 adult patients diagnosed with sepsis were screened during the study period. Among them, 39 patients were excluded due to the absence of CT imaging or the inability to measure the IVC diameter. Ultimately, 396 patients were included in the analysis (Figure 2).

The 30-day all-cause mortality rate was 81 (20.5%). Non-survivors were significantly older than survivors (median age: 85 vs. 81 years; p=0.001) (Table 1). Among the initial vital signs, systolic blood pressure, diastolic blood pressure, and Glasgow Coma Scale were significantly lower, while respiratory rate was significantly higher in non-survivors. No statistically significant differences were observed in comorbidities between groups. Urinary tract and gastrointestinal infections were significantly more common among survivors. Median levels of C-reactive protein and white blood cell counts did not differ significantly. However, markers of organ dysfunction and serum lactate levels were significantly elevated in non-survivors.

**Table 1:** Comparison of the characteristics of survivors and non-survivors Values are presented as mean±SD, median (IQR), or n (%), as appropriate. p-values were calculated using the Student’s t-test or Mann–Whitney U test for continuous variables and the chi-square test for categorical variables. Test statistic values are shown in the second-last column. P/F ratio: PaO_2_ (arterial oxygen tension)/FiO_2_ (fraction of inspired oxygen), COPD: chronic obstructive lung disease, CKD: chronic kidney disease, CRP: C-reactive protein, WBC: white blood cell, SOFA: Sequential (Sepsis-related) organ failure assessment, IVC: inferior vena cava, CT: computed tomography; AP/T: anteroposterior-to-transverse

Variables	Survivors (n=315) (Number, %)	Non-survivors (n=81) (Number, %)	Reference range values (where applicable)	Test statistic	p value
Age (years)	81 (74-87)	85 (81-89)		U=9765	0.001
Men	201 (64.0)	53 (65.4)		χ²=0.006	0.940
Initial vital signs					
Systolic blood pressure (mmHg)	131.8±32.1	123.7±33.2	90-140	t=-2.01	0.045
Diastolic blood pressure (mmHg)	73.1±18.4	66.7±19.1	60-90	t=-2.75	0.006
Heart rate (beats/min)	100 (86-111)	107 (91-121)	60-100	U=10305	0.008
P/F ratio	305 (222-390)	196 (76-305)		U=7459	<0.001
Respiratory rate (breaths/min)	20 (18-24)	24 (20-30)	12-20	U=9853	<0.001
Glasgow Coma Scale	14 (13-15)	14 (11-15)	15	U=9495	<0.001
Comorbidities					
Hypertension	128 (40.8)	40 (49.4)		χ²=1.957	0.162
Diabetes mellitus	76 (24.3)	15 (18.5)		χ²=1.203	0.273
COPD	13 (4.1)	5 (6.2)		χ²=0.612	0.434
Heart failure	19 (2.9)	8 (9.9)		χ²=1.480	0.224
Liver cirrhosis	5 (1.6)	1 (1.2)		χ²=0.055	0.814
CKD with renal replacement	16 (5.1)	3 (3.7)		χ² = 0.272	0.602
Origin of infection				χ² = 20.38	P<0.001
Respiratory	137 (43.6)	47 (58.0)			
Urinary	54 (17.2)	6 (7.4)			
Gastrointestinal	73 (23.2)	7 (8.6)			
Others	50 (15.9)	21 (25.9)			
Laboratory findings					
Total bilirubin (mg/dL)	1.0 (0.4-1.2)	0.7 (0.6-1.8)	0.2-1.2	U = 9204	<0.001
Creatinine (mg/dL)	1.1 (0.8-1.8)	1.4 (1.0-2.7)	0.6-1.0	U=10432	0.013
CRP (mg/dL)	8.4 (3.4-19.1)	11.1 (3.7-21.7)	<0.3	U=11876	0.324
WBC (×10^3/μL)	12.1 (8.4-16.4)	13.4 (8.2-18.9)	3.5-9.0	U=12248	0.609
Hemoglobin (g/dL)	12.2 (10.6-14)	12 (10.6-13.2)	13.0-17.0 (male); 11.5-15.0 (female)	U=11585	0.216
Platelet (×10^3/μL)	175 (125-240)	206 (150-268)	150-350	U=10709	0.031
Lactate (mmol/L)	1.7 (1.1-3.1)	3.2 (1.8-6.4)	0.5-2.0	U=7434	<0.001
SOFA score	4 (3-6)	6 (4-8)	0-24 (higher values indicate more severe illness)	U=8617	<0.001
Septic shock	15 (5)	22 (27)		χ²=12.28	0.002
AP/T ratio of IVC	0.525 (0.40-0.64)	0.401 (0.33-0.58)		U=9164	<0.001
IVC area (mm^2^)	245 (184-357)	225 (158.8-330)		U=9663	0.122
Positive airway pressure	5 (1.5)	7 (8.6)		χ²=3.40	0.079
Time to CT scan (min)	81.6 (71-92)	81.5 (70-93)		U=11576	0.214
Volume in 24 hours (ml)	2910 (2710-3120)	3360 (2830-3900)		U=11810	0.290

Importantly, the median IVC AP/T ratio was significantly lower in non-survivors compared to survivors (0.401 vs. 0.525; p<0.001). The median time from hospital arrival to CT scan did not significantly differ between groups (81.5 vs. 81.6 minutes; p=0.214). While detailed data on fluid or vasopressor administration prior to imaging were not available, CT was generally performed early after arrival, often before completion of full-volume resuscitation.

Multivariable logistic regression and diagnostic performance of the IVC AP/T ratio

To assess the association between the IVC AP/T ratio and 30-day mortality, we constructed a multivariable logistic regression model that included age and serum lactate level, together with five components of the SOFA score (total bilirubin, P/F ratio, platelet count, Glasgow Coma Scale, and serum creatinine) and the IVC AP/T ratio. In this primary model, the IVC AP/T ratio was not an independent predictor of mortality (odds ratio (OR): 0.210; 95% confidence interval (CI): 0.038-1.154; p = 0.073) (Table 2). In contrast, age (OR 1.038, 95% CI 1.009-1.067, p=0.011), lactate level (OR 1.263, 95% CI 1.144-1.395, p<0.001), and the P/F ratio (OR 0.994, 95% CI 0.992-0.997, p<0.001) were significantly associated with 30-day mortality.

**Table 2:** Multivariable logistic regression analysis to predict 30-day mortality Abbreviations: OR, odds ratio; CI, confidence interval; AP/T, anteroposterior-to-transverse. p-values were derived from logistic regression analysis. P/F ratio: PaO_^2^_ (arterial oxygen tension)/FiO_2_ (fraction of inspired oxygen), AP/T: anteroposterior/transverse, IVC: inferior vena cava.

Variables	Odds ratio (95% confidence interval)	p value
Age	1.038 (1.009-1.067)	0.011
Lactate	1.263 (1.144-1.395)	<0.001
Total bilirubin	1.018 (0.835-1.241)	0.857
P/F ratio	0.994 (0.992-0.997)	<0.001
Platelets	1.001 (0.974-1.029)	0.938
Glasgow Coma Scale	1.026 (0.925-1.138)	0.629
Creatinine	1.048 (0.892-1.231)	0.569
AP/T ratio of IVC	0.210 (0.038-1.154)	0.073

The AUROC for the IVC AP/T ratio in predicting 30-day mortality was 0.64. The optimal cutoff value, based on the maximal Youden index, was 0.44. At this threshold, the diagnostic accuracy was 63.5%, with a sensitivity of 61.7%, specificity of 68.6%, PPV of 33.6%, and NPV of 87.5% (Table 3).

**Table 3:** Diagnostic performance of IVC AP/T ratio (cutoff ≤0.44) for 30-day mortality IVC: Inferior vena cava, AP/T: anteroposterior/transverse, PPV: positive predictive value, NPV: negative predictive value

	Accuracy	Sensitivity	Specificity	PPV	NPV
AP/T ratio of IVC ≤ 0.44	63.5%	61.7%	68.6%	33.6%	87.5%

Comparison by AP/T cutoff (≤0.44 vs >0.44)

Patients were stratified into two groups based on the IVC AP/T ratio cutoff of 0.44: a "flattened IVC" group (≤0.44) and a "non-flattened IVC" group (>0.44) (Table 4). The flattened IVC group had higher heart rates (103 vs. 98 bpm; p<0.001). Although the median Glasgow Coma Scale score was similar between groups (14.0), the interquartile range differed, indicating a wider distribution and lower scores among non-survivors (13.0-15.0 vs. 12.0-14.0, p<0.01). No significant differences were observed in comorbidity profiles. However, the flattened IVC group had higher hemoglobin levels (12.7 g/dL vs 11.8 g/dL; p=0.015), lower platelet counts (173×10³/μL vs. 199×10³/μL; p=0.014) and higher serum lactate levels (2.4 mmol/L vs. 1.8 mmol/L; p<0.001). The median SOFA score did not differ significantly between the groups. Nevertheless, the flattened IVC group had a significantly higher prevalence of septic shock (14.1% vs. 6.5%; p=0.020) and a higher 30-day mortality rate (33.6% vs. 12.6%; p<0.001). There were no significant differences in the proportion of patients receiving positive pressure ventilation during CT or in the time from admission to CT imaging. The volume of intravenous fluids administered within 24 hours was significantly greater in the flattened IVC group (2,500 mL vs. 2,200 mL; p=0.035).

**Table 4:** Comparison of patients stratified by the IVC AP/T ratio cutoff Values are presented as mean±SD, median (interquartile range (IQR)), or n (%), as appropriate. p-values were calculated using the Student’s t-test or Mann–Whitney U test for continuous variables and the chi-square test for categorical variables.  Test statistic values are shown in the second-last column. P/F ratio: PaO^2^ (arterial oxygen tension) / FiO_2_ (fraction of inspired oxygen), COPD: Chronic obstructive lung disease, CKD: chronic kidney disease, CRP: C-reactive protein, WBC: white blood cell, SOFA: Sequential (Sepsis-related) organ failure assessment, IVC: inferior vena cava, AP/T: anteroposterior/transverse, CT: computed tomography

Variables	Flattened (n=149) (Number, %)	Non-flattened (n=247) (Number, %)	Reference range (where applicable)	Test statistic	p value
Age (years)	82 (73-88)	83 (79-87)		U=16853	0.147
Men	97 (65.1)	158 (64.0)		χ²=0.520	0.820
Initial vital signs					
Systolic blood pressure (mmHg)	129.1±32.2	130.8±32.6	90-140	t=-0.481	0.631
Diastolic blood pressure (mmHg)	70.7±19.6	72.3±18.1	60-90	t=-0.721	0.471
Heart rate (beats/min)	103 (91-118)	98 (84-110)	60-100	U=14710	<0.001
P/F ratio	276 (137-390)	295 (206-390)		U=16753	0.135
Respiratory rate (breaths/min)	22 (18-26)	20 (18-24)	12-20	U=16774	0.136
Glasgow Coma Scale	14 (12-15)	14 (14-15)	15	U=15283	0.003
Comorbidities					
Hypertension	68 (45.6)	100 (40.5)		χ²=1.001	0.315
Diabetes mellitus	34 (22.8)	57 (23.1)		χ²=0.007	0.936
COPD	6 (4.0)	12 (4.9)		χ²=0.148	0.700
Heart failure	8 (5.4)	19 (7.7)		χ²=0.790	0.374
Liver cirrhosis	2 (1.3)	4 (1.6)		χ²=0.048	0.827
CKD with renal replacement	8 (5.4)	11 (4.5)		χ²=0.171	0.680
Origin of infection				χ²=4.064	0.397
Respiratory	65 (43.6)	120 (48.6)			
Urinary	20 (13.4)	40 (16.2)			
Gastrointestinal	35 (23.5)	45 (18.2)			
Others	29 (19.5)	42 (17.0)			
Laboratory findings					
Total bilirubin (mg/dL)	0.9 (0.5-1.5)	0.9 (0.6-1.7)	0.2-1.2	U=17011	0.295
Creatinine (mg/dL)	1.2 (0.8-2.4)	1.2 (0.8-1.8)	0.6-1.0	U=17755	0.558
CRP (mg/dL)	9.5 (2.3-21.4)	8.8 (3.6-18.7)	<0.3	U=18123	0.853
WBC (×10^3/μL)	12.5 (8.4-19.6)	11.3 (8.4-19.6)	3.5-9.0	U=16661	0.115
Hemoglobin (g/dL)	12.7 (11.1-14.4)	11.8 (10.4-13.5)	13.0-17.0 (male); 11.5-15.0 (female)	U=15728	0.015
Platelet (×10^3/μL)	199 (144-261)	173 (118-235)	150-350	U=15628	0.014
Lactate (mmol/L)	2.4 (1.4-4.6)	1.8 (1.1-3.0)	0.5-2.0	U=14218	<0.001
SOFA score	5 (3-7)	4 (3-6)	0-24 (higher scores indicate more severe illness)	U=16268	0.051
Septic shock	21 (14.1)	16 (6.5)		χ²=7.775	0.020
IVC area (mm^2^)	175 (131-222)	295 (220-399)		U=5525	<0.001
Positive airway pressure	6 (4.0)	6 (2.4)		χ²=0.794	0.373
Time to CT scan (min)	59 (37-97)	64 (40-100)		U=17545	0.547
Volume in 24 hours (ml)	2500 (1800-4350)	2200 (1700-3200)		U=15943	0.035
30-day mortality	50 (33.6)	31 (12.6)		χ²=25.0	<0.001

Among the 396 patients included in the analysis, only eight (2.0%) had missing values in at least one covariate. The missing data were confined to lactate, total bilirubin, platelet count, and mortality, while all other covariates in the regression model were complete. Given the small proportion of missingness and the absence of apparent systematic patterns, we assumed the data were missing at random (MAR) and used complete-case analysis as the primary approach. To assess the robustness of our findings, we also performed multiple imputation using chained equations (20 imputations). The imputed model demonstrated a statistically significant association between the IVC AP/T ratio and 30-day mortality (OR=1.77, 95% CI: 1.08-3.46, p=0.040), suggesting that the prognostic impact of this marker may be more clearly observed when accounting for potential bias introduced by missing data.

The linearity assumption between each continuous covariate and the log-odds of 30-day mortality was evaluated using locally weighted scatterplot smoothing (LOWESS). Although slight curvatures were noted in some variables, they were considered acceptable based on visual inspection, their limited deviation from linearity, and clinical plausibility. Therefore, all continuous variables were retained in their original scale to maintain model simplicity and interpretability. We further explored potential effect modification by introducing interaction terms into the multivariable logistic regression model. The prognostic effect of the IVC AP/T ratio remained statistically significant (p=0.010). Additionally, several interaction terms reached statistical significance, including age×lactate, age×IVC AP/T, PF ratio×IVC AP/T, and GCS×IVC AP/T. These findings suggest that the impact of lactate levels and IVC flattening on mortality may be more pronounced in older patients or in those with respiratory or neurological compromise. 

Multicollinearity was assessed using variance inflation factors (VIFs) in the complete-case dataset (n=388). All covariates had VIF values below 1.25 (mean VIF=1.14), indicating negligible multicollinearity in the regression model. Model calibration was assessed using the Hosmer-Lemeshow goodness-of-fit test (χ²=6.11, p=0.635), indicating good agreement between the observed and predicted outcomes. The model showed moderate discriminative performance (Pseudo R²=0.2116), and several clinically relevant predictors - including age, serum lactate, and PF ratio - were statistically significant. The association between the IVC AP/T ratio and 30-day mortality demonstrated a marginal trend (p=0.073). To assess the robustness of our findings, we performed bootstrapped logistic regression using 1,000 replications. The direction and magnitude of the associations for age, serum lactate, and P/F ratio remained consistent and statistically significant. To assess the robustness of the logistic regression model, we performed bootstrap resampling with 1,000 iterations using in-hospital mortality as the binary outcome. The direction and magnitude of the associations for age, serum lactate, and P/F ratio remained consistent and statistically significant. However, the association between the IVC AP/T ratio and 30-day mortality, while showing a similar effect size, was no longer statistically significant after bootstrapping (OR=0.21, 95% CI: 0.04-1.15, p=0.135), reflecting increased uncertainty due to the limited sample size and potential variance in model stability.

## Discussion

To our knowledge, this study is among the first to elucidate the association between the IVC AP/T ratio measured on CT and sepsis as a patient outcome. Therefore, this novel finding is considered worthy of reporting. The present study demonstrated that stratifying sepsis patients based on a CT-derived IVC AP/T diameter ratio cutoff value of 0.44 was associated with a significantly higher 30-day mortality in the lower-ratio group. Patients with a flattened IVC (AP/T≤0.44) had a mortality rate of 33.6%, compared to 12.6% in those with a more rounded IVC (p<0.001). Several studies have examined IVC morphology as a marker of fluid status, primarily using ultrasonography. Feissel et al. [13] and Zhang et al. [14] demonstrated that respiratory variation in IVC diameter predicts fluid responsiveness in critically ill patients. More recently, Preau et al. [12] reported diagnostic accuracy of IVC collapsibility in sepsis. However, these ultrasound-based approaches are limited by operator dependency. In contrast, Kim et al. [18] showed that CT-derived IVC ratios were associated with outcomes in septic shock, but their study was restricted to shock patients. Our findings extend these observations by demonstrating that the AP/T ratio on routine CT is associated with prognosis across a broader sepsis population, supporting its potential utility in general emergency settings. These findings suggest that IVC flattening, as assessed via CT, may reflect intravascular volume depletion and serve as a potential risk-stratification marker. Although not statistically significant in the multivariable model - likely due to residual confounding or limited sample size - the point estimate suggested a possible association that warrants external validation.

Volume resuscitation remains a cornerstone of sepsis management [3,23]. Increasing preload can improve cardiac output, thereby supporting organ perfusion and oxygen delivery [24]. However, excessive fluid administration has been associated with adverse outcomes [25], and clinicians frequently face difficulties in estimating circulating plasma volume and fluid requirements [26]. Vital signs may be unreliable due to measurement errors or medication effects, and laboratory tests are influenced by numerous variables. Central venous pressure is not predictive of outcomes [25,27]. While dynamic indices such as stroke volume responsiveness provide useful information, they require invasive monitoring that may be unavailable in many settings. Thus, no standardized algorithm exists for evaluating volume status or guiding fluid therapy in sepsis. This is especially challenging in elderly patients and those with comorbidities. Although ultrasound measurement of the IVC can assist in assessing volume status, it is highly operator-dependent and subject to limitations such as obesity, bowel gas, and anatomical variance. In contrast, CT imaging - routinely performed in patients with sepsis to identify infection sources or other pathologies - offers an objective and reproducible method to evaluate IVC morphology. Importantly, this can be done concurrently during standard diagnostic workup without additional equipment. Given its accessibility and quantifiability, CT-based IVC assessment could be a pragmatic adjunct for assessing volume status and estimating prognosis, especially in non-specialist or resource-limited settings. Given the modest AUROC observed, the predictive capacity of IVC morphology alone may be limited. However, when integrated with established sepsis severity indices such as the SOFA score, it could potentially enhance clinical risk stratification. Notably, the PPV was relatively low at 33.6%, whereas the NPV was high at 87.5%. These characteristics suggest that the AP/T ratio may be more useful in ruling out high-risk cases rather than definitively identifying them. Therefore, this metric should not be interpreted as a stand-alone predictor of mortality but rather as a supplementary tool to assist in early triage and clinical decision-making alongside established prognostic indices.

This study has several limitations. First, it was a single-center retrospective analysis, which may limit the generalizability of the findings. Second, patient compliance with breath-holding instructions during CT imaging was not verified, which may have introduced variability in IVC diameter measurements. Third, confounding variables such as intrathoracic pressure, intra-abdominal pressure, valvular heart disease, and pulmonary hypertension were not controlled, all of which could influence IVC morphology independently of volume status. Fourth, although total 24-hour fluid volumes were available, detailed data on the volume of resuscitation administered prior to CT were not recorded. This limitation is critical, as early fluid administration may affect IVC morphology and introduce potential reverse causation; sicker patients may have received more fluids, blunting the association between IVC shape and mortality. Fifth, measurements were derived from 5-mm CT slices, which may be susceptible to partial-volume effects and introduce measurement uncertainty. This is particularly relevant in evaluating vascular structures such as the IVC; future studies using thinner slices or high-resolution reconstruction would improve measurement fidelity. Sixth, the study population was predominantly elderly (median age >80 years), potentially limiting the applicability of the findings to younger or more diverse sepsis populations. Seventh, due to the modest sample size, we did not perform propensity score matching to adjust for residual confounding. Finally, as with all observational studies, causality cannot be inferred from the observed associations.

## Conclusions

In conclusion, this single-center retrospective study demonstrated that a CT-derived IVC AP/T ratio cutoff of 0.44 stratified sepsis patients into groups with significantly different mortality risks. Although the ratio was not independently predictive in multivariable analysis, its simplicity, objectivity, and availability during routine CT imaging suggest that it may serve as a pragmatic adjunct for early risk assessment. Given its modest predictive accuracy, the AP/T ratio should not be used in isolation but rather considered alongside established prognostic indices such as the SOFA score. Larger prospective, multicenter studies are warranted to validate these findings and to clarify whether incorporating CT-based IVC assessment into sepsis care can meaningfully improve patient outcomes. Future research should also aim to standardize CT imaging protocols, formally assess inter-rater reliability, and evaluate the additive prognostic value of the AP/T ratio when combined with existing clinical scores.
